# Recombinant spider silk protein eADF4(C16)-RGD coatings are suitable for cardiac tissue engineering

**DOI:** 10.1038/s41598-020-65786-4

**Published:** 2020-05-29

**Authors:** Johannes P. M. Kramer, Tamara B. Aigner, Jana Petzold, Kaveh Roshanbinfar, Thomas Scheibel, Felix B. Engel

**Affiliations:** 1 Experimental Renal and Cardiovascular Research, Department of Nephropathology, Institute of Pathology, Friedrich-Alexander-Universität Erlangen-Nürnberg (FAU), Schwabachanlage 12, 91054 Erlangen, Germany; 2 Lehrstuhl Biomaterialien, Prof.-Rüdiger-Bormann Straße 1, 95447 Bayreuth, Germany; 3 Bayreuther Zentrum für Kolloide und Grenzflächen (BZKG), Bayerisches Polymerinstitut (BPI), Bayreuther Zentrum für Molekulare Biowissenschaften (BZMB), Bayreuther Materialzentrum (BayMAT), Universitätsstraße 30, Universität Bayreuth, Bayreuth, D-95447 Germany; 4 MURCE, Muscle Research Center Erlangen, Erlangen, Germany

**Keywords:** Bioinspired materials, Biomaterials - cells, Biomedical materials, Cardiology

## Abstract

Cardiac tissue engineering is a promising approach to treat cardiovascular diseases, which are a major socio-economic burden worldwide. An optimal material for cardiac tissue engineering, allowing cardiomyocyte attachment and exhibiting proper immunocompatibility, biocompatibility and mechanical characteristics, has not yet emerged. An additional challenge is to develop a fabrication method that enables the generation of proper hierarchical structures and constructs with a high density of cardiomyocytes for optimal contractility. Thus, there is a focus on identifying suitable materials for cardiac tissue engineering. Here, we investigated the interaction of neonatal rat heart cells with engineered spider silk protein (eADF4(C16)) tagged with the tripeptide arginyl-glycyl-aspartic acid cell adhesion motif RGD, which can be used as coating, but can also be 3D printed. Cardiomyocytes, fibroblasts, and endothelial cells attached well to eADF4(C16)-RGD coatings, which did not induce hypertrophy in cardiomyocytes, but allowed response to hypertrophic as well as proliferative stimuli. Furthermore, Kymograph and MUSCLEMOTION analyses showed proper cardiomyocyte beating characteristics on spider silk coatings, and cardiomyocytes formed compact cell aggregates, exhibiting markedly higher speed of contraction than cardiomyocyte mono-layers on fibronectin. The results suggest that eADF4(C16)-RGD is a promising material for cardiac tissue engineering.

## Introduction

In 2016, cardiovascular disease (CVD) was responsible for approximately 17.6 million deaths worldwide, an increase of 14.5% compared to 2006. CVD may cause myocardial infarction, which results in the loss of functional myocardium and scar formation and can lead to chronic heart failure. As the human heart does not macroscopically regenerate after damage, cardiac function decreases^1^. Various approaches have been utilized to compensate for lost cardiomyocytes due to cell death, including stem cell therapy, activation of endogenous stem cells, induction of cardiomyocyte proliferation, and cardiac tissue engineering^2–4^. Zimmerman *et al*. were able to significantly increase the function of infarcted hearts in immune-suppressed rats upon implantation of grafts consisting of cardiomyocytes embedded in a mixture of type I collagen and Matrigel^5^, a gelatinous protein mixture secreted by Engelbreth-Holm-Swarm (EHS) mouse sarcoma cells consisting mainly of laminin, nidogen, collagen and heparan sulfate proteoglycans. The potential of cardiac tissue engineering to improve heart function after myocardial infarction has since been validated by several independent groups in small as well as large animal models^6–8^. At the same time, clinical trials using engineered cardiac patches in humans have been performed, and their safety could be confirmed. While Menasché *et al*. proved the technical feasibility to graft fibrin patches containing human embryonic stem cell-derived cardiac progenitor cells onto hearts of heart failure patients^9^, it remains unclear if this procedure will improve heart function in heart failure patients.

Several materials, including collagen^10^, fibrin^9^, gelatin^11^, and silk^12,13^ have been utilized for cardiac tissue engineering. Silk materials have several advantages, since they can be processed into various morphologies, they provide sufficient mechanical strength, they are biocompatible, present low levels of inflammatory response, and are noncytotoxic^13–18^. Previously, we have shown that materials made of silk fibroin of the silkworm *Antheraea mylitta* allow better cardiomyocyte attachment compared to materials made of *Bombyx mori* silk fibroin due to the presence of an intrinsic RGD-sequence^12^, which is also present in fibronectin^19^, a natural component of the extracellular matrix of several organs^20^, including the heart^21,22^, on which neonatal rat cardiomyocytes have been shown to attach^23^. However, natural materials such as *Antheraea mylitta* silk show a highly variable quality due to factors such as living conditions and feeding, making their silk unsuitable for clinical applications^15^. In contrast, recombinant technologies allow to produce silk proteins with highest and continuous quality. Previously, it has been shown that eADF4(C16) can be recombinantly produced at large scale with high yields and high purity^24,25^. The sequence of eADF4(C16) is based on 16 repeats of the consensus sequence (C) of the European garden spider’s (*Araneus diadematus*) fibroin 4. eADF4(C16) can be further modified, allowing the protein to be tailored to specific applications^26,27^.

As cells do not attach well to eADF4(C16)^28^, the aim of this study was to evaluate the interaction of cardiomyocytes with RGD-functionalized eADF4(C16) coatings. For this purpose, we assessed whether cardiomyocytes attach to eADF4(C16)-RGD coatings, respond to stimulation with hypertrophic and proliferative factors, and determined beating properties. This is important, even though it has previously been shown that several other cell types attach to eADF4(C16)-RGD coatings^29^, because cardiomyocytes exhibit a particular adhesion behavior and generally attach poorly to most surfaces. This property is routinely used to enrich cardiomyocytes by pre-plating^12^. Moreover, we have recently observed that cardiomyocytes attach better to eADF4(κ16) than non-cardiomyocytes^13^. In addition, eADF(C16)-RGD has several advantages over other engineered spider silk variants: i) Cardiomyocytes attach poorly to eADF(C16)^13^. ii) eADF(C16)-RGD but not eADF(κ16) can be 3D printed^30,31^. iii) As cardiomyocytes attach poorly to eADF(C16) coatings^13^ and RGD-modifications have no influence on eADF4(C16) coating properties^29^, eADF(C16)-RGD most likely enables cardiomyocyte attachment via integrins in the same manner as natural substrates, such as fibronectin^21^. In contrast, eADF(κ16) enables attachment via its polycationic character, which may result in very different intracellular signaling processes.

## Results

### Properties of eADF4(C16)-RGD coatings

**Figure 1:**
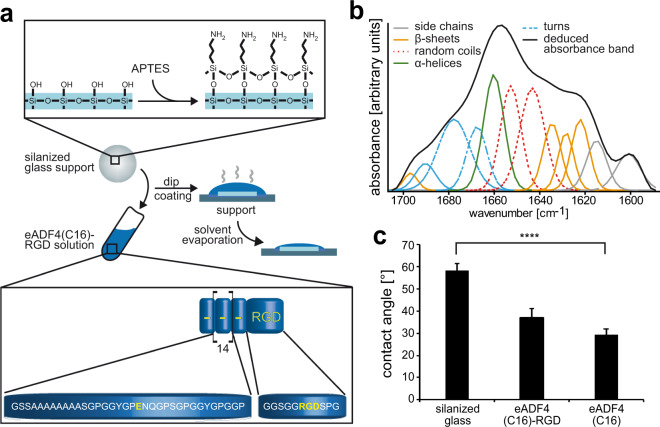
eADF4(C16)-RGD coating processing and characterization. (**a**) First, glass was silanized using (3-aminopropyl)triethoxysilane (APTES) to render the surface charge positive. Then, silanized glass coverslips were dip-coated in spider silk solution and dried on a non-adhesive support. The spider silk protein was engineered based on the consensus sequence of the repetitive core domain of *Araneus diadematus* fibroin 4 (ADF4). The achieved C-module was repeated 16 times in the recombinant protein. Further, an RGD-tag was included C-terminally in the protein, yielding eADF(C16)-RGD. (**b**) Absorbance spectrum and Fourier self-deconvolution of the amide I band of an eADF4(C16)-RGD coating on glass. Black line: deduced absorbance band, others: deconvoluted secondary structure contributions according to published values. (**c**) Contact angle measurements. Contact angles were determined using the sessile drop method. n = 20, data are mean ± SD. ****p < 0.001.

Due to the inherent negative surface charge of cells and hence preferential attachment to polycationic surfaces^13^, the polyanionic nature of eADF4(C16) and its lack of cell binding sequences prohibits cell attachment. Since an integrin-binding RGD motif has been shown to promote cardiomyocyte attachment^12^, we investigated cardiac cell behavior on coatings made of eADF4(C16)-RGD, a variant of eADF4(C16) in which an RGD-tag is fused C-terminally^29^. Glass coverslips were dip-coated into a solution of eADF4(C16)-RGD in formic acid (Fig. 1a), applying 0.15 mg/cm^2^ silk protein as previously published^13^. As the surfaces of both eADF4(C16)-RGD and silicate glass^32^ are negatively charged, electrostatic repulsion and hence delamination of the coatings occurred. Therefore, glass coverslips were silanized using (3-aminopropyl)triethoxysilane (APTES). Hoechst 33342 staining was used to confirm successful coating as previously described^13^, showing a blue background illumination (Supplementary Fig. 1). No obvious signs of degradation were observed during the experiments. This is in agreement with previous studies investigating the stability of silk films and coatings^33–35^.

Spider silk coatings prepared from formic acid typically possess thermodynamically stable β-sheet-rich structures making them water-insoluble^36,37^, and this secondary structure could be confirmed using spectroscopy and successive analysis of the amide I band according to Hu *et al*.^38^ using Fourier self-deconvolution (FSD) (Fig. 1b) with 22.53 ± 0.25% beta-sheets, 14.71 ± 0.32% alpha helices, 31.83 ± 1.34% random coils, and 24.83 ± 0.99% turns. As expected, silanized glass showed no signal in the amide I region of FTIR spectra. Next, surface hydrophilicity of eADF4(C16)-RGD coatings was determined in comparison to that of eADF4(C16) coatings and silanized glass using water contact angle measurements (Fig. 1c). Both eADF4(C16)-RGD and eADF4(C16) coatings were more hydrophilic than silanized glass (contact angle: 58.4° ± 3.2°) with contact angles of 37.2° ± 3.9° and 29.5° ± 2.4°, respectively (p < 0.001).

### eADF4(C16)-RGD coatings are suitable for cardiac cell attachment

To evaluate eADF4(C16)-RGD coatings for cardiac cell adhesion, cells from postnatal day 3 (P3) rat hearts were isolated and cultured on eADF4(C16)-RGD coatings. Fibronectin coatings were used as positive control, as fibronectin is a natural component of the cardiac extracellular matrix^21,22^ to which neonatal rat cardiomyocytes have been shown to attach^23^. Cells were cultured in the presence of fetal bovine serum (FBS), as serum withdrawal has been associated with reduced cardiomyocyte cell viability in culture^39^. Yet, it is important to examine the interactions between coatings and cardiomyocytes without or at low concentrations of serum, as it has been demonstrated that FBS induces hypertrophy in neonatal rat cardiomyocytes^13^ and human pluripotent stem cell-derived cardiomyocytes^40^.

**Figure 2:**
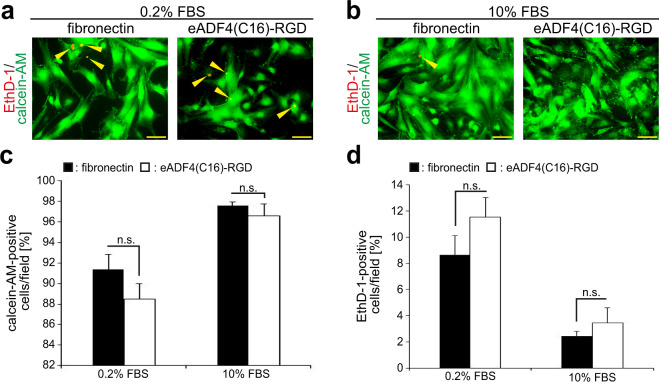
Cardiac cells attach to eADF4(C16)-RGD coatings. Cardiac cells were cultured for 48 h with 0.2% FBS and 10% FBS on fibronectin and eADF4(C16)-RGD coatings. Calcein-AM-positive cells (green) indicate live cells, while EthD-1-positive cells (red) indicate dead cells (yellow arrows). (**a**,**b**) Calcein-AM/EthD-1 analysis. (**c**,**d**) Quantitative analysis of calcein-AM -positive and as well as EthD-1 positive cells per miscroscopic field (0.1 mm^2^). n = 3, data are mean ± SD. n.s.: statistically not significant. Scale bars: 50 µm.

In order to confirm that eADF4(C16)-RGD coatings are nontoxic to cardiac cells, cardiac cells were allowed to attach overnight to eADF4(C16)-RGD and fibronectin coatings, were cultured for 48 h in the presence of 0.2% and 10% FBS (v/v), and a calcein-acetoxymethylester (calcein-AM) and Ethidium-homodimer-1 (EthD-1) live/dead assay was conducted (Fig. 2a,b). Analysis of the data revealed that both coatings were non-toxic, showing a low level of EthD-1 positive cells. In addition, there was no statistically significant difference between the percentage of live calcein-AM- or dead EthD-1-positive cells cultured on eADF4(C16)-RGD or fibronectin coatings at 0.2% or 10% FBS (Fig. 2c,d) suggesting that eADF4(C16)-RGD coatings are suitable for the adhesion of cardiac cells.

**Figure 3:**
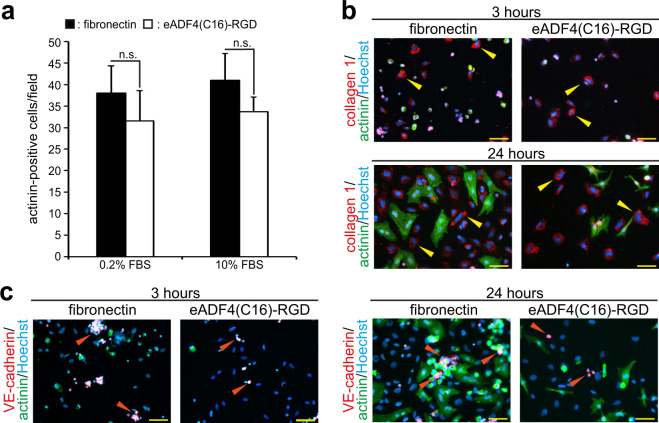
Cardiomyocyte, fibroblast and endothelial cell attachment to eADF4(C16)-RGD coatings after 3 h and 24 h, or 48 h of incubation. Cardiac cells were incubated in 0.2% FBS and 10% FBS. Cardiomyocytes were visualized by staining for sarcomeric α-actinin (green) and DNA (Hoechst, blue). (**a**) Quantitative analysis of the average number of actinin-positive cells per microscopic field after incubation on the spider silk coating for 48 h. n = 3, data are mean ± SD. n.s.: statistically not significant. (**b**) Cells were positive for collagen-1 staining (red, fibroblasts), as marked by yellow arrows. (**c**) Cells were positive for VE-cadherin (red, endothelial cells), as indicated by orange arrows. Scale bars: 50 µm.

**Figure 4:**
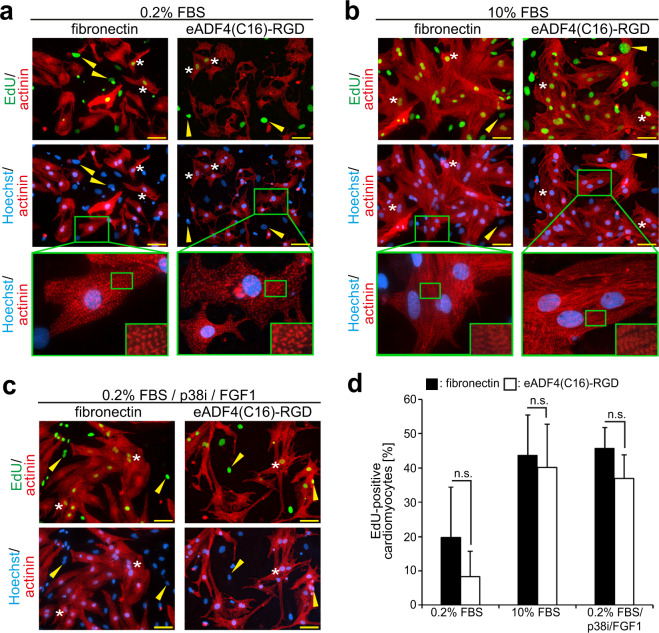
Cardiomyocytes cultured on eADF4(C16)-RGD coatings respond properly to proliferative stimuli and exhibit well-differentiated sarcomeres. Cardiomyocytes were cultured with 0.2% FBS, 10% FBS or FGF1/p38i/0.2% FBS, and stained for EdU-incorporation, indicating DNA synthesis. (**a**-**c**) Cardiomyocytes were stained for sarcomeric α-actinin (red), EdU (green) and DNA (blue). White asterisks indicate EdU-positive cardiomyocytes, while yellow arrowheads point to EdU-positive non-myocytes. Green boxes show zoom-in cutouts of cardiomyocyte sarcomere structures. (**d**) Quantitative analysis of the percentage of EdU-positive cardiomyocytes per microscopic field (0.1 mm^2^). n = 3, data are mean ± SD. n.s.: statistically not significant. Scale bars: 50 µm.

To determine if cardiomyocytes attach to eADF4(C16)-RGD coatings, isolated and enriched cardiomyocytes were allowed to attach overnight to eADF4(C16)-RGD and fibronectin coatings and were cultured for 48 h in the presence of 0.2% and 10% FBS (v/v). Subsequently, cells were fixed, stained for the cardiomyocyte-specific marker sarcomeric α-actinin, and the number of cardiomyocytes per microscopic field was counted (Fig. 3a, for representative images see Fig. 4). Analysis of this data showed that cardiomyocytes attached to eADF4(C16)-RGD coatings, and there was no significant difference between the number of actinin-positive cells between eADF4(C16)-RGD and fibronectin coatings or between different serum conditions for either surface. Our data demonstrate that eADF4(C16)-RGD coatings support the attachment of cardiac cells as well as coatings made of fibronectin, a natural component of the cardiac extracellular matrix^21,22^.

Cardiac tissue contains a variety of cells, including cardiomyocytes, fibroblasts and endothelial cells^41^. It has been shown that the presence of non-myocytes in cardiac patches improves the structure and function of tissue^42^ and, furthermore, vascularization is required for the survival of a functional patch^43^. Thus, cardiac cells were isolated without cardiomyocyte enrichment, allowed to attach for 3 h or 24 h in the presence of 0.2% or 10% FBS and stained for cell-type-specific markers (actinin, cardiomyocytes; collagen-1, fibroblasts; VE-cadherin, endothelial cells^13^) and DNA. As shown in Fig. 3, all investigated cardiac cell types attached to fibronectin and eADF4(C16)-RGD coatings. Note, fibroblasts and especially endothelial cells were less frequent than cardiomyocytes. Thus images in Fig. 3 are not representative in regard to cardiomyocyte attachment. In addition, induction of hypertrophy (increase in cell size) of cardiomyocytes by fibronectin gives at a first glance the impression of a difference even at the same cell density.

Taken together, these data demonstrate that eADF4(C16)-RGD coatings are comparable to coatings made of fibronectin, a natural component of the cardiac extracellular matrix^21,22^, with regards to cardiac cell adhesion and cell viability.

### Cardiomyocytes cultured on eADF4(C16)-RGD coatings respond to proliferative stimuli

For a cardiac patch to contract properly and thus improve heart function, the presence of a high density of cardiomyocytes is essential. One way to increase cardiomyocyte density after engineering a cardiac tissue is the induction of cardiomyocyte proliferation. Therefore, the response to proliferative stimuli of cardiomyocytes cultured on eADF4(C16)-RGD was assessed using an EdU-incorporation assay (Fig. 4). In this assay, the nucleotide analog EdU is added to the medium and thus can be utilized by the cells during DNA synthesis. This allows visualization of cells undergoing DNA synthesis via fluorescent labelling and the subsequent detection of EdU incorporated in the DNA via fluorescence microscopy.

Cardiomyocytes were allowed to attach overnight to the coatings and were then cultured for 48 h in the presence of 0.2% FBS, 10% FBS, or a mixture composed of fibroblast growth factor 1 (FGF1), mitogen-activated protein kinase p38i inhibitor (p38i) and 0.2% FBS. Previously, it has been shown that FGF-1/p38i induces proliferation^44^ and 10% FBS promotes cell cycle re-entry in neonatal cardiomyocytes^45^. Quantitative analysis demonstrated that 10% FBS, as well as FGF1/p38i/0.2% FBS, induced DNA synthesis in cardiomyocytes attached to eADF4(C16)-RGD or fibronectin coatings, with no statistically significant difference between the two coatings (Fig. 4d). In addition, as observed previously for neonatal rat cardiomyocytes cultured on gelatin^44,46,47^ and eADF4(κ16)^13^, cardiomyocytes stimulated with FGF1/p38i/0.2% FBS presented an elongated morphology on eADF4(C16)-RGD coatings (Fig. 4C). These data indicate that cardiomyocytes behave similarly on eADF(C16)-RGD coatings as on the inert material gelatin, and that eADF4(C16)-RGD coatings allow attached cardiomyocytes to properly respond to proliferative stimuli.

### Cardiomyocytes cultured on eADF4(C16)-RGD coatings respond to hypertrophic stimuli

**Figure 5:**
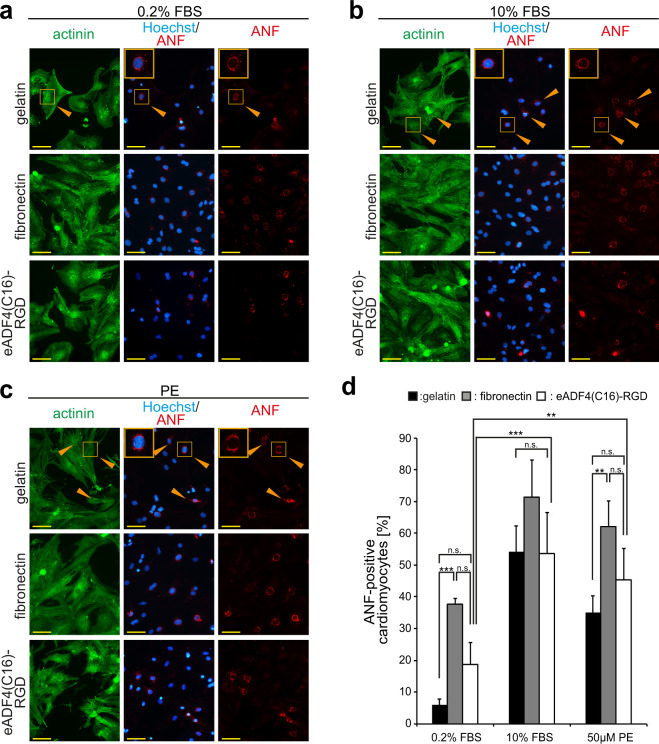
Cardiomyocytes respond to hypertrophic stimuli on eADF(C16)-RGD coatings as well as control substrates. Cardiomyocytes were stimulated using 0.2% FBS, 10% FBS or 50 × 10^−6^ M phenylephrine (PE) and stained for atrial natriuretic factor (ANF) to assess the hypertrophic response of cardiomyocytes on the indicated coatings. (**a**–**c**) Cardiomyocytes were stained for sarcomeric α-actinin (green), ANF (red) and DNA (Hoechst, blue). Orange arrows and boxes highlight examples of hypertrophic cardiomyocytes. (**d**) Quantitative analysis of the percentage of ANF-positive cardiomyocytes per microscopic field (0.1 mm^2^). n = 4, data are mean ± SD. n.s.: statistically not significant. *p < 0.05. **p < 0.02. ***p < 0.01).

*In vivo*, cardiomyocytes display a physiological hypertrophic response, i.e. an increase in cellular size, upon stimuli such as hormones or growth factors released during times of increased cardiovascular stress, e.g. in athletes or during pregnancy^48^. Previously, it has been shown that fibronectin exhibits a hypertrophic effect on neonatal rat cardiomyocytes in culture^49^. Notably, cardiomyocytes stimulated with 0.2% FBS or FGF1/p38i/0.2% FBS appeared smaller on eADF4(C16)-RGD coatings than on fibronectin coatings (Fig. 4a,c), suggesting that eADF4(C16)-RGD coatings may have no hypertrophic effect on cardiomyocytes. In order to evaluate whether eADF4(C16)-RGD coatings indeed exhibit no hypertrophic effect and whether cardiomyocytes cultured on eADF4(C16)-RGD coatings are able to respond properly to strong (10% FBS) and weak (phenylephrine, PE) hypertrophic stimuli^13^, cells were cultured for 48 h in the presence of these factors, and 0.2% FBS was used as a control. Gelatin, an established material for tissue engineering^11^, was used as a control substrate^12,13,50^, since fibronectin could not be used due to hypertrophic effects on neonatal rat cardiomyocytes in culture^49^ and associated pathophysiological hypertrophy *in vivo*^21^. Cells were stained for atrial natriuretic factor (ANF), which is expressed around the nucleus in hypertrophic cardiomyocytes^51^ and has been successfully used as a marker for cardiomyocyte hypertrophy^13^ (Fig. 5).

Our data show that the number of ANF-positive cardiomyocytes stimulated with 0.2% FBS is not significantly different on eADF4(C16)-RGD coatings compared to that on gelatin (Fig. 5d). In addition, stimulation of cardiomyocytes cultured on eADF4(C16)-RGD coatings with 10% FBS or 50 µM PE resulted in a significant increase in ANF-positive cardiomyocytes comparable to that on the other matrices (Fig. 5d). These data indicate that eADF4(C16)-RGD coatings exhibit no significant hypertrophic effect and allow cardiomyocytes to respond adequately to hypertrophic stimuli.

### Cardiomyocytes cultured on eADF4(C16)-RGD coatings exhibit normal beating behavior

**Figure 6:**
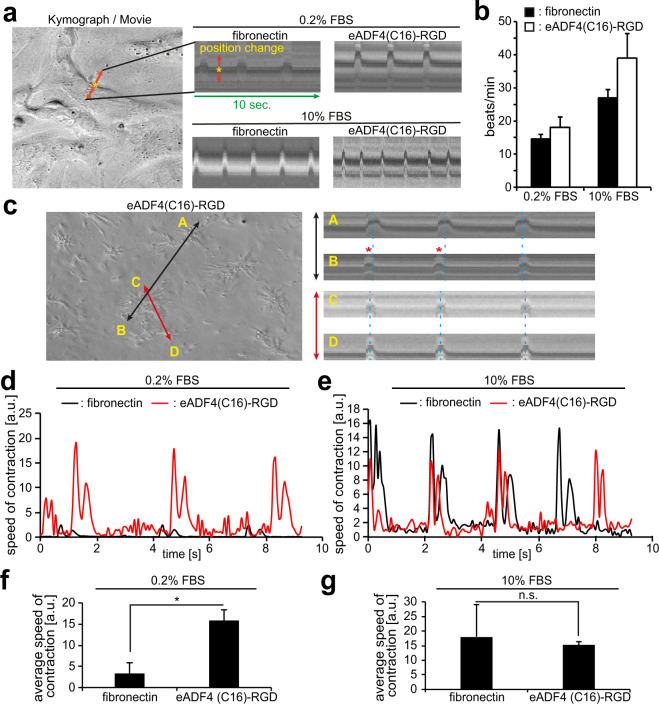
Cardiomyocytes cultured on eADF4(C16)-RGD coatings exhibit proper contractility. (**a**) Kymograph images showing regular cardiomyocyte contraction patterns with increased beating frequency at 10% FBS compared to 0.2% FBS. (**b**) Quantitative analysis of cardiomyocyte contraction on spider silk and fibronectin coatings in beats per minute. (**c**) Kymographs comparing beating synchronicity between neighboring clusters of cardiomyocytes grown on eADF4(C16)-RGD coatings. Left: the black line indicates two distant cardiomyocyte clusters (yellow letters A and B) and the red line indicates two immediately adjoining clusters (yellow letters C and D). Right: corresponding Kymographs; Note the asynchronicity between distant clusters (A and B, red asterisks and blue dotted lines do not align), as well as synchronicity between immediately adjoining clusters (C and D, blue dotted lines align). (**d**,**e**) Examples of MUSCLEMOTION analysis of speed of contraction in the presence of 0.2% FBS (**d**) and 10% FBS (**e**). (**f**,**g**) Quantitative analysis of (**d**) and (**e**). n = 3, data are mean ± SD. *p < 0.05. n.s.: statistically not significant.

Naturally, for a bioengineered cardiac patch to perform well *in vivo*, cardiomyocytes must beat regularly on/within the scaffold. Kymograph software (Fiji) was used to determine the number of beats per minute of cultured cardiomyocytes by measuring beating cardiomyocytes’ membrane deflection in 10-second videos (Fig. 6a). Example videos are included as part of the supplementary material (Supplementary Movie 1–4). Analysis of the videos indicated that cardiomyocytes cultured on eADF4(C16)-RGD coatings beat regularly (Fig. 6a), with no statistically significant difference between eADF4(C16)-RGD and fibronectin coatings. Yet, there was a trend towards a higher beating frequency on eADF4(C16)-RGD coatings (Fig. 6b). These data suggest that cardiomyocytes cultured on eADF4(C16)-RGD coatings exhibit normal beating behavior.

A closer analysis of the videos revealed that cardiomyocytes grown on eADF4(C16)-RGD coatings tend to cluster or aggregate. Kymograph analysis showed that these clusters do not all beat synchronously (Fig. 6c). Finally, MUSCLEMOTION analyses of the videos demonstrated a higher speed of contraction for cardiomyocytes cultured on eADF4(C16)-RGD coatings at low serum conditions compared to those on fibronectin coatings (Fig. 6d), which was statistically significant (Fig. 6f). This suggests that cardiomyocytes cultured on eADF4(C16)-RGD coatings exhibit improved contractility (see also Discussion). Notably, speed of contraction for cardiomyocytes cultured on eADF4(C16)-RGD coatings was not improved at high serum concentrations (Fig. 6d–g). In contrast, speed of contraction for cardiomyocytes cultured on fibronectin coatings was markedly improved reaching the levels observed for eADF4(C16)-RGD coatings. This indicates that cardiomyocytes cultured on eADF4(C16)-RGD coatings are less dependent on external stimuli in order to maintain basic contractile properties. This may allow a more constant contractile performance in future cardiac tissue engineering applications. All other measured parameters showed no significant differences between eADF4(C16)-RGD and fibronectin coatings (Supplementary Fig. 2) and no obvious differences were observed in cell eccentricity, cell shape, sarcomere alignment, and sarcomere density. These data indicate that eADF4(C16)-RGD allows cardiomyocytes to exhibit proper beating behavior.

## Discussion

We conclude that the recombinant spider silk protein eADF4(C16)-RGD is a promising material for the application in cardiac tissue engineering and offers the many benefits of silk materials, such as low immunogenicity and biodegradability^33,34^. Cell types vital for cardiac tissue engineering, cardiomyocytes, endothelial cells, and fibroblasts, attach in a similar manner to eADF4(C16)-RGD and fibronectin coatings. Although fibronectin is a component of the native cardiac extracellular matrix^20,23^, fibronectin is not optimal for cardiac tissue engineering as it contributes to pathological cardiac hypertrophy but not physiological growth^21^. Coatings made of eADF4(C16)-RGD are noncytotoxic without obvious pharmacological properties, and do not hinder cardiomyocytes to respond adequately to extracellular stimuli. Finally, cardiomyocytes form compact cellular aggregates on eADF4(C16)-RGD coatings. One highly interesting feature of eADF4(C16)-RGD, which provides an outlook for future studies, is its 3D printability by robotic dispensing without requiring crosslinking additives or thickeners for mechanical stabilization^30,31^. Investigation on fabrication of scaffolds for clinical application using bioinks based on eADF4(C16)-RGD and stem cell-derived cardiomyocytes will be the next steps to evaluate putative applications.

In recent years, major developments have been made in the field of cardiac tissue engineering based on mouse model results indicating that engineered cardiac patches can improve cardiac function after myocardial infarction^5^ leading to first clinical safety trials^4,9^. Yet, several hurdles have to be overcome to establish a therapy. One major issue is that current artificial constructs do not exhibit the force measured in human adult heart muscle^52^, despite identifying several parameters improving force development such as mechanical strain/load, chronic electric pacing, and supplementation of medium with L-thyroxin^53^. This might be due to the fact that the utilized cells still do not reach an adult-like phenotype and the inability of generating thick, vascularized cardiac tissue in which cells are hierarchically organized. Several approaches are currently used to address this problem such as the use of prefabricated scaffolds (decellularized matrices or matrices generated in a non-cell-compatible manner) and 3D printing^53^. Yet, these approaches are currently limited by a low number of available materials. Our data, in conjunction with previously published data, suggest that eADF4(C16)-RGD is a promising material for coating prefabricated scaffolds as well as 3D printing in cardiac tissue engineering.

In order to generate a functional cardiac patch, materials are required that allow attachment not only of cardiomyocytes, but also of non-myocytes, which are known to improve the structure and function of engineered cardiac tissues^42^. Furthermore, vascularization is necessary for patch survival^43^. Here, we show that fibroblasts and endothelial cells adhere to eADF4(C16)-RGD coatings. In addition, eADF4(C16)-RGD coatings appeared to exert no obvious effect on cardiomyocyte function. Importantly, cardiomyocytes grown on eADF4(C16)-RGD coatings responded to hypertrophic stimuli. This suggests that cardiac patches based on eADF4(C16)-RGD will respond to extracellular stimuli *in vivo* to adapt heart function to altered physiological conditions during periods of increased strain, such as in athletes or during pregnancy^48^.

The generation of thick cardiac constructs, which are densely packed with cardiomyocytes, remains a major challenge due to for example challenges in seeding prefabricated scaffolds. A solution could be the promotion of cardiomyocyte proliferation^54^, which could compensate for initially low cardiomyocyte densities. Here, we show that cardiomyocytes grown on eADF4(C16)-RGD coatings responded properly to an inducer of cardiomyocyte proliferation^44^. In addition, we observed that cardiomyocytes on eADF4(C16)-RGD coatings agglomerate to compact cellular structures. This might be a very useful property in generating a more compact cardiac tissue construct. Yet, in this study, local compaction was associated with light arrhythmia. Further studies will show if this hampers the functionality and whether this can for example be compensated by the inclusion of electroconductive materials^10,55^.

Kymograph and MUSCLEMOTION video analyses show that cardiomyocytes grown on eADF4(C16)-RGD or fibronectin coatings exhibit a similar contractile behavior. However, cardiomyocytes exhibited a significantly higher speed of contraction on eADF4(C16)-RGD than on fibronectin coatings when cultured at low serum concentrations. Cardiac contractility is discernible by the maximum contraction speed and its ejection fraction^56^. A higher speed of contraction therefore translates directly to improved cardiac function. In addition, a trend, albeit not statistically significant, was observed towards a faster relaxation time of cardiomyocytes on eADF4(C16)-RGD coatings. The speed at which ventricular cardiomyocytes are able to undergo relaxation is of vital importance for the modulation of the heart rate *in vivo*. If, e.g. due to stimulation by the sympathetic nervous system, the heart beat is sped up in healthy humans, the length of the diastole is shortened more radically compared to that of the systole relative to the length of the cardiac cycle^57^.

Taken together, our data suggest that the recombinant spider silk protein eADF4(C16)-RGD is a promising material for cardiac tissue engineering. Future studies will show whether the 3D printability of eADF4(C16)-RGD together with the use of cardiomyocytes and non-myocytes derived from human induced pluripotent stem cells allow the generation of cardiac patches for clinical application.

## Methods

### Preparation of recombinant silk protein

Engineered *Araneus diadematus* fibroin 4 (C16) with an RGD tag (eADF4(C16)-RGD) comprises 16 repeats of a C-module (sequence: GSSAAAAAAAASGPGGYGPENQGPSGPGGYGPGGP) and an RGD tag (GGSGGRGDSPG) for integrin binding^29^. The recombinant silk protein was produced and purified as described previously^58^.

### Silanization procedure

Glass coverslips (Ø 12 mm, Thermo Fisher Scientific) were silanized according to Verné *et al*.^59^ using (3-aminopropyl)triethoxysilane (APTES, sigma aldrich). Glass coverslips were cleaned for 5 min in acetone in an ultrasound bath, quickly washed with H_2_O_MQ_, followed by another acetone, water and acetone treatment. After this washing procedure, the silanization reaction was carried out using APTES in ethanol (0.2 ml APTES in 50 ml ethanol p.a.) for 5 h under gentle movement. The coverslips were then washed twice with 100% (v/v) ethanol before a thermal treatment at 100 °C for 1 h was performed to consolidate bonding of the glass surface with silane. Directly before coating, the coverslips were washed again with H_2_O_MQ_ and ethanol.

### Spider silk coating procedure

The silanized glass coverslips were coated as described previously^13^. The lyophilized spider silk protein eADF4(C16)-RGD was dissolved in formic acid and diluted with H_2_O_MQ_ (5:1, formic acid:H_2_O_MQ_; silk concentration 0.34% w/v) before the coverslips were dip-coated in the eADF4(C16)-RGD solution and allowed to dry on Parafilm M.

### Water contact angle measurements

A Surftens-universal (OEG GmbH, Germany) in combination with SCA-20 software was used to determine the water contact angle of silanized- and silk-covered glass coverslips. Uniform drops were deposited and the contact angles were determined after equilibration at room temperature using the sessile drop method. 20 measurements were done (n = 20) per sample.

### Attenuated total reflection-Fourier transform infrared (ATR-FTIR) spectroscopy

A Bruker Tensor 27 spectrometer (Bruker, Germany) equipped with a Ge-crystal was used to record ATR-FTIR spectra of eADF4(C16)-RGD coatings on glass, and silanized glass was used as a control. The spectra are accumulations of 100 scans from 4000–800 cm^−1^ with a resolution of 4 cm^−1^. Fourier self-deconvolution (FSD) was performed to determine the secondary structure content by analyzing the amide I region (1595–1705 cm^−1^) according to Hu *et al*.^38^ Five samples were analyzed using the Opus software (Bruker, Germany) (n = 5).

### Neonatal rat cardiomyocyte isolation and cell culture

All experiments were executed in accordance to the Guide for the Care and Use of Laboratory Animals Directive 2010/63/EU, European Parliament. Organ extraction and primary cell culture preparation was approved by the local Animal Ethics Committee of Erlangen, conforming to governmental and international regulations on animal experimentation (protocol TS-9/16 Nephropatho). Ventricular cardiomyocyte isolation was performed as previously published^13^. In brief, ventricles from 3 days-old (P3) Sprague Dawley rats were digested using the gentleMACS Dissociation kit (Miltenyl Biotec, 130–098–373) according to the manufacturer’s instructions. Afterwards, cardiomyocytes were enriched by pre-plating for 90 min at 37 °C. The suspension of non-attached cells enriched in cardiomyocytes was collected, centrifuged for 5 min at 330 × g, resuspended in DMEM F12 + GLUTAMAX containing 100 U/mg/ml penicillin/streptomycin and cultured in medium containing supplements: 3.0 mM Na-pyruvate (Sigma, S8636), 2.0 mM L-glutamine (Invitrogen, 25030081), 0.1 mM ascorbic acid (Sigma A4034), 1:200 insulin/transferrin/Na-selenite supplement (Sigma A4034), and 0.2% bovine serum albumin (BSA, Sigma A7409), if not stated otherwise. For the assessment of non-myocyte attachment, cardiac cells were isolated as described by Sadoshima *et al*. with minor modifications^60^. Ventricular cardiac cells from P3 Sprague Dawley rats were isolated utilizing 0.14 mg/ml collagenase II (Gibco 17101-015) and 0.5 mg/ml pancreatin (Sigma P3292). Isolated cells were cultured without preplating in the above-described supplemented medium. Cells were seeded at a density of 150,000/500 µl/well on glass coverslips (Ø 12 mm, Thermo Fisher Scientific) in 24-well tissue culture plates. For video analysis experiments, a cell density of 100,000/250 µl/well was seeded in Thermo Scientific Nunc Lab-Tek II CC2 8-well chambers. For proliferation assays, cells were seeded in DMEM F12 + GLUTAMAX containing 100 U/mg/ml penicillin/streptomycin and 1% fetal bovine serum (FBS).

### Fibronectin and gelatin coating procedure

Prior to coating, glass coverslips were sterilized twice with 70% ethanol and once with 100% ethanol for 5 min each, then left to dry under UV-light for 30 min. For fibronectin coating, coverslips were incubated with 100 µl of 25 µg/ml fibronectin (Sigma-Aldrich, F1141)/ Dulbecco’s Phosphate-Buffered Saline (DPBS, Gibco, 14190-094) solution for 2 h at 37 °C. Coverslips were coated with gelatin by adding 500 µl of 1% gelatin (Sigma)/water solution and incubation at 37 °C for 2 h. The solution was then removed, fresh gelatin solution was added once again, and coverslips were incubated for another 2 h. Finally, after removing the solution, coverslips were incubated for another hour under the same conditions to dry.

### Immunofluorescence staining

Unless otherwise stated, cells were fixed using 3.7% paraformaldehyde (PFA) solution/PBS for 20 min, washed 3× with PBS for 5 min, permeabilized for 10 min using 0.5% Triton X, and blocked for 20 min in blocking buffer (5% BSA and 0.2% Tween in PBS). Primary antibodies were diluted in blocking buffer and applied for 1.5 h at room temperature: mouse sarcomeric α-actinin (abcam, ab9465) 1:250, rabbit collagen-1 (Rockland, 600-401-103-0) 1:250, rabbit VE cadherin (abcam, ab33168) 1:200, and rabbit ANF (Phoenix Pharmaceuticals, H-005-24) 1:500. Secondary antibodies were diluted 1:500 and applied for 45 min at room temperature: Alexa Fluor donkey anti-mouse 488 nm (Invitrogen, A21202), Alexa Fluor donkey anti-mouse 594 nm (Invitrogen, A21203), Alexa Fluor donkey anti-rabbit 594 nm (Invitrogen, A21207). For EdU incorporation assays, the Click-iT EdU Alexa Fluor 488 Imaging Kit (Life Technologies, C10337) was used. DNA and silk attachment were visualized using Hoechst 33342 (Invitrogen, C10337) at a dilution of 1:5000 in PBS (final: 2 µg/ml). Subsequently to staining, coverslips were mounted using Fluoromount G (Invitrogen, 00-4958-2). Images and videos were acquired using a Keyence BZ9000 Fluorescence Microscope (Keyence, Osaka, Japan). Images were edited using Adobe Photoshop, and overlays created and analyzed (unless otherwise stated) using the Keyence software “BZ-II Analyzer”.

### Live/dead cell viability assay

Cells were seeded in DMEM F12 + GLUTAMAX + 100 U/mg/ml penicillin/streptomycin + supplements and were allowed to attach overnight. They were then washed once with DPBS, and the medium was changed, supplemented with either 0.2% FBS or 10% FBS. After incubation for 48 h, cells were washed three times in DPBS for 5 min and incubated with calcein-AM (0.25 µl/ml) in PBS and ethidium-homodimer 1 (1 µl/ml) in PBS (Life Technologies, L2334) for 10 min at 37 °C and 5% CO_2_. Subsequently, cells were fixed in 3.7% PFA for 15 min, washed in DPBS, mounted and fixed on the slides using nail polish to allow immediate microscopy. The percentage of calcein- and ethidium-homodimer-positive cells per microscopic field was assessed for 10 randomly chosen microscopic fields (0.1 mm²) per experiment.

### Cell adhesion

Cells were allowed to attach for 3 h and 24 h (cardiac cells without preplating) or 48 h (enriched cardiomyocytes). Subsequently, cells were stained for DNA, sarcomeric α-actinin, collagen 1, or for DNA, sarcomeric α-actinin, and VE cadherin. To determine the average number of attached cardiomyocytes per microscopic field, experimental data from the performed hypertrophy assay (see below) were used to assess the average number of actinin-positive cardiomyocytes per microscopic field for 10 randomly chosen microscopic fields (0.1 mm²) per experiment.

### Hypertrophy assay

Cells were seeded in DMEM F12 + GLUTAMAX + 100 U/mg/ml penicillin/streptomycin + supplements and were allowed to attach overnight. They were then washed with DPBS, and the medium was changed, supplemented with either 0.2% FBS, 10% FBS, or 50 µM PE (phenylephrine, Sigma Aldrich, P6126). After another 48 h, the cells were fixed and stained to determine the average percentage of atrial natriuretic factor (ANF)-positive cardiomyocytes per microscopic field for 10 randomly chosen microscopic fields (0.1 mm²) per experiment.

### Proliferation assay

Cells were seeded in DMEM F12 + GLUTAMAX + 100 U/mg/ml penicillin/streptomycin + 1% FBS without supplements and allowed to attach overnight. After washing with DPBS, the medium was changed to DMEM F12 + GLUTAMAX + 100 U/mg/ml penicillin/streptomycin + 0.2% FBS, + 10% FBS, or + 0.2% FBS, 50 ng/ml FGF1 (R&D Systems, 132-FA) and 10 µM p38i (Tocris, SB203580-HCl). To the corresponding wells, additional p38i (10 µM) was added every 24 h for the next 48 h. 5-ethynyl-2’-deoxyuridine (EdU, Life Technologies, C10337) was added after the medium change after 24 h and 48 h to yield a concentration of 30 µM/well. 72 h after the medium was changed, cells were fixed and stained using the kit according to the manufacturer’s protocol. Subsequently, the average percentage of EdU-positive cardiomyocytes per microscopic field was determined for 10 randomly chosen microscopic fields (0.1 mm²) per experiment.

### Video analysis

Cells were seeded in DMEM F12 + GLUTAMAX + 100 U/mg/ml penicillin/streptomycin + supplements and incubated overnight. They were then washed with DPBS, and the medium was changed to medium containing 0.2% FBS or 10% FBS. After 48 h, the cells were washed, the medium was changed once again with the respective FBS concentrations and incubated overnight. Then, for a period of 10 seconds, the number of beats during this time was either counted or videos recorded and the number of beats was determined using the Fiji Kymograph-plugin four times per experiment. Briefly, when a line segment is drawn on a video file, the Kymograph-plugin can generate a time-series image from the video’s constituent images that the line’s coordinates refer to. In the resulting images, the x-axis represents time with the width of the image covering the entire 10-second period or a portion of the video, while the y-axis represents the axis of movement. MUSCLEMOTION analyses of beating movies of cardiomyocytes was done based on a previously published method.^61^

### Statistical analysis

n is the number of independent experiments. Each independent experiment was performed with 2 technical replicates. 10 randomly chosen microscopic fields (0.1 mm^2^) from each 2 technical replicates were chosen, meaning 30 fields when n was 3, and used for quantitative analyses. Data are expressed as the mean ± SD of at least three independent experiments. The statistical significance of the differences between means was evaluated by a two-tailed Student’s t-test (Microsoft Excel) or where appropriate by one way ANOVA followed by Bonferroni’s post-hoc test (IBM SPSS Statistics Version 21). p < 0.05 was considered statistically significant.

## Supplementary information

Supplementary Information.

Supplementary Information 2.

Supplementary Information 3.

Supplementary Information 4.

Supplementary Information5.
