# Supplementary material for: Recombinant spider silk protein eADF4(C16)-RGD coatings are suitable for cardiac tissue engineering
Source: Sci Rep. 2020 May 29;10:8789. doi: 10.1038/s41598-020-65786-4 (PMC7260369; doi:10.1038/s41598-020-65786-4)

Supplementary information for

Recombinant spider silk protein eADF4(C16)-RGD coatings are suitable for cardiac tissue engineering

Johannes P. M. Kramer, Tamara B. Aigner, Jana Petzold, Kaveh Roshanbinfar,

Thomas Scheibel, Felix B. Engel

Content:

1) Supplementary Figure Legends

2) Supplementary Movie Captions

3) Supplementary Figure 1

4) Supplementary Figure 2

5) Supplementary Movie 1

6) Supplementary Movie 2

7) Supplementary Movie 3

8) Supplementary Movie 4

Supplementary Figure Legends

Supplementary Figure 1: Validation of eADF4(C16)-RGD-coating. Hoechst 33342 staining was used to confirm successful coating of coverslips with eADF4(C16)-RGD, showing an apparent blue background illumination. Left: For demonstration, a coverslip coated with eADF4(C16)-RGD was scratched using tweezers, revealing the absence of background illumination in the now exposed glass (white asterisk). Red arrow: edges of scratched eADF4(C16)-RGD coatings. Right: background illumination of an intact eADF4(C16)-RGD coating.

Supplementary Figure 2: MUSCLEMOTION analysis. Acquired movies of cardiomyocytes cultured on fibronectin- or eADF4(C16)-RGD coatings were analyzed upon 0.2% or 10% FBS stimulation. (**a**) Relaxation time. (**b**) Contraction amplitude. (**c**) Peak-to-peak time. (**d**) Beating rates. n = 3. Data are mean ± SD. There was no statistically significant difference between materials.

Supplementary Movie Captions

Supplementary Movie 1: Cardiomyocytes cultured on fibronectin at 0.2% FBS.

Supplementary Movie 2: Cardiomyocytes cultured on fibronectin at 10% FBS.

Supplementary Movie 3: Cardiomyocytes cultured on eADF4(C16)-RGD at 0.2% FBS.

Supplementary Movie 4: Cardiomyocytes cultured on eADF4(C16)-RGD at 10% FBS.

Kramer J *et al.*, Supplementary Figure 1

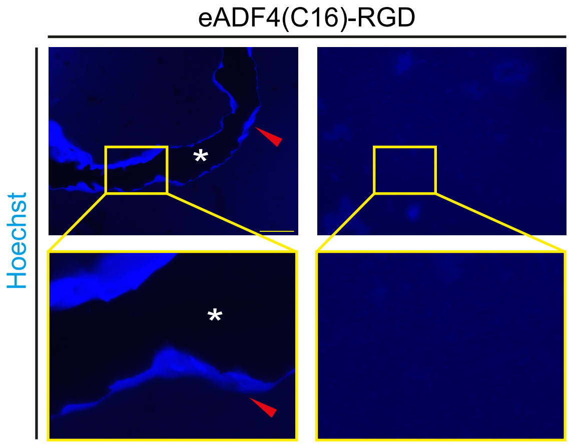

Kramer J *et al.*, Supplementary Figure 2

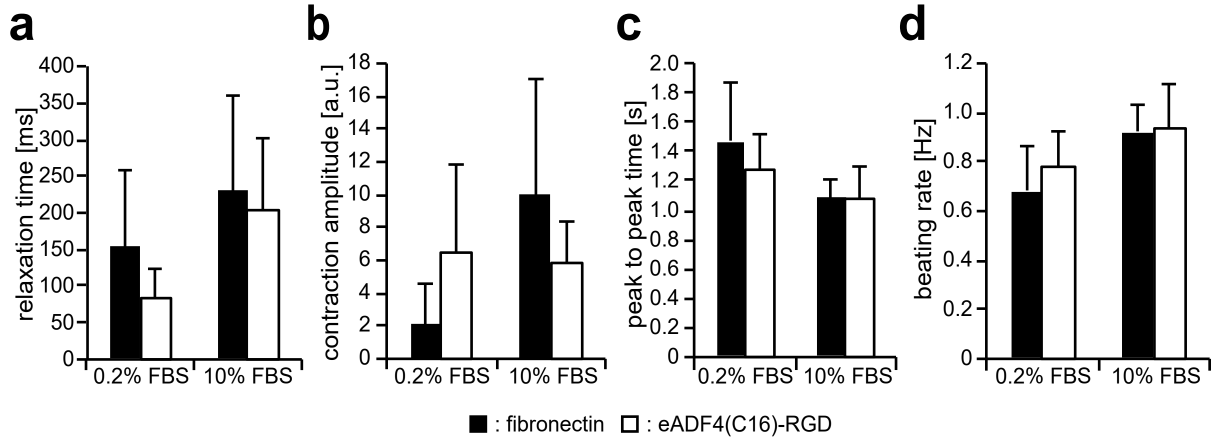

Supplement: Supplementary file 5 — Supplementary Information5. [file 41598_2020_65786_MOESM5_ESM.pdf]
